# Tobacco cessation intervention for individuals with severe mental illness in Bangladesh, India, and Pakistan: protocol for a multi-country feasibility randomised controlled trial (SCIMITAR-SA)

**DOI:** 10.1016/j.conctc.2026.101620

**Published:** 2026-02-21

**Authors:** Garima Bhatt, Kousar Ishaq, Aatik Arsh, Omara Dogar, Justin Fenty, Cath Jackson, Muqaddas Asif, Jothisha C, Shannon Halmkan, Fahmida Islam, Krishna Prasad Muliyala, Venkata Lakshmi Narasimha, Steve Parrott, Rebecca R, Fahmidur Rahman, Faraz Siddiqui, Heather Thomson, Han-I Wang, Rumana Huque, Imran B. Chaudhry, Asad Tamizuddin Nizami, Catherine Hewitt, Najma Siddiqi, Kamran Siddiqi, Simon Gilbody, Pratima Murthy

**Affiliations:** a Department of Health Sciences, University of York, York, UK; b Institute of Psychiatry, Rawalpindi Medical University, Rawalpindi, Pakistan; c Institute of Physical Medicine and Rehabilitation, Khyber Medical University Peshawar, Pakistan; d York Trials Unit, University of York, York, UK; e Valid Research Ltd, Lewes, UK; f Pakistan Institute of Living and Learning, Karachi, Pakistan; g Division of Population Health, Health Services Research and Primary Care, University of Manchester, Manchester, UK; h Department of Psychiatry, National Institute of Mental Health and Neurosciences (NIMHANS), Bengaluru, India; i ARK Foundation, Dhaka, Bangladesh; j Public Health, Leeds City Council, Leeds, UK; k Department of Psychiatry, Ziauddin University, Karachi, Pakistan; l Division of Psychology and Mental Health, School of Health Sciences, Faculty of Biology, Medicine and Health, University of Manchester, Manchester, UK; m Hull York Medical School, University of York, York, UK; n Bradford District Care NHS Foundation Trust, Bradford, UK

**Keywords:** Tobacco, Cessation, Mental health, South asia, Intervention

## Abstract

**Background:**

Tobacco is consumed by two-thirds of individuals with severe mental illness (SMI). Despite a high tobacco-related disease burden, there is a lack of evidence-based cessation interventions for individuals with SMI living in low- and middle-income countries. This study aims to evaluate the feasibility and acceptability of a culturally adapted behavioural intervention for tobacco cessation (SCIMITAR-SA) delivered in mental health services in Bangladesh, India, and Pakistan.

**Methods:**

A two-arm, parallel-group, individually randomised, multi-country feasibility trial will be conducted across six mental health facilities in urban centres. All trial participants will receive Very Brief Advice (VBA) and an educational leaflet from their clinical team. Additionally, those in the intervention arm will receive up to seven structured behavioural support sessions. Salivary cotinine and anabasine will be used to biochemically verify abstinence at seven months post-randomisation. Quantitative outcomes will assess feasibility of conducting a definitive trial, including recruitment and retention rates, session attendance, completeness of baseline assessments and outcome measures at four and seven months and use of health resources. An embedded process evaluation will explore the feasibility and acceptability of trial processes, and of the delivery and receipt of the VBA and SCIMITAR-SA interventions. Economic outcomes will assess the feasibility of collecting cost and resource-use data to inform a future definitive trial.

**Discussion:**

The SCIMITAR-SA trial will provide essential evidence on the feasibility of delivering culturally adapted cessation support for people with SMI in South Asia and inform scalable integration into routine psychiatric care across low- and middle-income settings.

**Registration:**

ISRCTN registry (ISRCTN91038721)

## 1 Introduction

Mental disorders are a major contributor to the global disease burden, ranking among the top ten causes of health loss worldwide [1]. Individuals with Severe Mental Illness (SMI) (Schizophrenia, Schizoaffective disorder, Psychosis, Bipolar illness and Severe Depression with/without psychosis) represent one of the most vulnerable population groups [2]. They face pronounced health disparities, physical multimorbidity [3], higher mortality, shorter life expectancy, driven by both biological and behavioural factors such as smoking, poor diet, drug use, and inactivity [[3], [4], [5]]. Tobacco consumption is a major modifiable cause of morbidity and mortality, resulting in more than seven million deaths annually and contributing to prolonged disability and suffering from tobacco-related conditions [6].

Around two in three individuals with SMI are current smokers [7] and reducing tobacco use in this group is recognised as an effective strategy to narrow the life expectancy gap [8]. South Asian LMICs, particularly Bangladesh, India, and Pakistan bear the highest burden of tobacco-related disease, encompassing both smoking and smokeless tobacco (SLT) [[9], [10], [11]]. While data from the region are limited, smoking prevalence as high as 50% among individuals with SMI [12], and widespread use of SLT in the general population [13] is reported. Despite population-level declines in tobacco use over the past four decades, little reduction has occurred among individuals with SMI [14,15]. A multi-country survey in Bangladesh, India, and Pakistan reported higher tobacco use among people with SMI, especially men, yet only 38.4% had received advice to quit [16].

Although individuals with SMI are often motivated to improve their physical health, including quitting tobacco, they require tailored cessation support due to complex treatment regimens, higher dependence, and the need for psychotropic medication adjustment, differing literacy levels, cognitive difficulties, medication affecting self-efficacy and motivation [[17], [18], [19]].

Evidence from high-income countries, notably the UK SCIMITAR trial, has demonstrated that SMI-specific interventions significantly increase quit rates, improve quality of life, and are cost-effective [17].

However, these findings cannot be directly translated to LMICs, where tobacco consumption varies and includes non-cigarette products (bidi and hookah) and SLT products, and where health system capacities, cultural norms, and resource contexts differ substantially [[18], [19], [20], [21]]. No large-scale, contextually adapted cessation trials for individuals with SMI exist in these settings, representing a critical evidence gap [22,23]. Conducting a feasibility trial is therefore essential to assess intervention acceptability, relevance, and operational practicality of its delivery, and to enable refinement of our approach to its integration within the diverse contexts before evaluating effectiveness in a definitive trial [[24], [25], [26]]. The Smoking Cessation Intervention for Severe Mental Ill Health–South Asia (SCIMITAR-SA) has been adapted from the UK National Centre for Smoking Cessation and Training (NCSCT) Standard Treatment Programme [27], drawing on evidence from two South Asian cessation trials, IMPACT4S (smoking cessation support for people with SMI) [28] and ASTRA (SLT users) [29], and expanded to support dual tobacco users. Adaptation involved community engagement and involvement workshops, pre-testing with individuals with SMI and intervention delivery staff, and stakeholder consultations across Bangladesh, India, and Pakistan to guide context-specific modifications. SCIMITAR-SA follows a patient-centred approach, tailoring support and promoting autonomy in culturally aligned cessation choices, and aims to address this gap through a feasibility randomised controlled trial across Bangladesh, India, and Pakistan to lay a robust groundwork for a future definitive trial and strengthen broader efforts toward tobacco control in this population.

### 1.1 Aim of the study

We aim to conduct a feasibility randomised controlled trial of SCIMITAR-SA to inform the design and implementation of a future definitive trial for tobacco cessation in mental health settings of three South Asian countries.

### 1.2 Research questions for the study

1. Is it feasible to identify individuals with SMI who use tobacco, recruit them to a trial of tobacco cessation conducted in mental health facilities and retain them for up to seven months?
2. Is it feasible to collect data for the primary (seven months biochemically-verified continuous abstinence) and secondary outcomes of a potential full scale trial and for the economic evaluation?
3. Is it feasible to deliver SCIMITAR-SA intervention in mental health facilities?
4. Is SCIMITAR-SA intervention acceptable to participants, and feasible to deliver by tobacco dependence advisors and mental health facility staff in its current form, or does it need further adaptation and refinement?

## 2 Methods

The feasibility trial protocol is prospectively registered (ISRCTN91038721) and adheres to SPIRIT checklist and CONSORT guidelines [30].

### 2.1 Study design

A two arm, parallel group, individually randomised, multi-country, multicentre, external pilot trial of the SCIMITAR-SA intervention will be conducted, with an embedded qualitative process evaluation and a preliminary economic evaluation.

### 2.2 Study settings

The feasibility trial will be conducted across six mental health facilities in three South Asian countries: two in Bangladesh (National Institute of Mental Health, Dhaka; Thengamara Mohila Sabuj Sangha (TMSS) Medical College, Bogura), one in India (National Institute of Mental Health and Neuro Sciences (NIMHANS), Bengaluru), and three in Pakistan (Civil Hospital,Karachi, Karwan-e-Hayat, Karachi, and Institute of Psychiatry, Rawalpindi Medical University, Rawalpindi). Sites were selected based on research infrastructure, availability of trained mental health staff, managerial engagement, and sufficient numbers of individuals with SMI.

### 2.3 Study participants and eligibility criteria

Adults (≥18 years) with a confirmed diagnosis of SMI, (schizophrenia, schizoaffective disorder, bipolar affective disorder, psychosis, or severe depression with/without psychosis) and who are current users of smoked and/or smokeless tobacco and are willing to quit all forms of tobacco use, will be recruited. Participants will be identified from outpatient departments of the six participating mental health facilities. Eligible participants must be clinically stable, as assessed by the treating psychiatrist (no electroconvulsive therapy or neurostimulation in the preceding three months and <25% change in medication dosage), reside within the facility's catchment area, and provide informed consent. Inclusion requires self-reported daily or near-daily tobacco use (smoked and/or smokeless) for at least six months and ≥25 days in the past month [28], willingness to quit all forms of tobacco within 30 days, and ability to attend up to seven face-to-face counselling sessions. Only one participant per household will be enrolled to avoid risk of contamination. Exclusion criteria are minimal and include self-reported use of e-cigarettes, heated tobacco products, or oral nicotine pouches in the past 30 days and/or in combination with either smoking and/or smokeless forms of tobacco; comorbid drug or alcohol use; personality, eating, neurodevelopmental or post-traumatic stress disorders; receipt of pharmacological and/or behavioural cessation support (beyond brief advice) within the previous 30 days; and dual users who are willing to quit only one form of tobacco.

### 2.4 Details of interventions

The details of each are given below. All participants with SMI will receive usual care, including Very Brief Advice (VBA) and a self-help educational leaflet. Participants allocated to the intervention arm will, in addition, receive the SCIMITAR-SA intervention.

### 2.5 Very Brief Advice

VBA, developed by the UK National Centre for Smoking Cessation and Training, is a three-step evidence-based approach comprising Ask (about their tobacco use), Advise (about quitting), and Act (to prompt quit attempts through available cessation resources) [31,32]. VBA has been adapted from the ASTRA smokeless tobacco cessation trial [33]. It will include a brief conversation on tobacco use status (smoked and/or smokeless), advice to quit and providing a self-help educational leaflet [34]. VBA will be delivered by health professionals during outpatient visits in interactions lasting up to 1 min. Staff involved in SMI patient care will receive orientation on VBA delivery. As a low-intensity intervention, VBA satisfies ethical duty-of-care requirements while minimising contamination of the active intervention arm.

### 2.6 SCIMITAR-SA intervention

SCIMITAR-SA intervention consists of up to seven individual counselling sessions (20–30 min each) delivered over approximately six to seven weeks by trained Tobacco Dependence Advisors (TDAs) experienced in caring for mental health patients (specialist or non-specialist) who receive specific SCIMITAR-SA training and supervision. The intervention will allow for two pre-quit sessions, a quit day session (corresponding with 30 days since enrollment) and weekly follow up to the 4-week post-quit point. In line with the findings of SCIMITAR-UK [35], the sessions may be adapted into shorter, more frequent contacts, with duration extending beyond 6-7 weeks as needed. A model of trainer-of-trainers (ToT) will supervise and support TDAs, with both groups receiving comprehensive training and follow-up discussion from behaviour change experts.

Sessions will be delivered face-to-face up to the quit day, with the option of remote delivery (phone or WhatsApp) if in-person meetings are not feasible. The intervention adopts a participant-centred approach, helping participants articulate reasons for quitting, identify triggers, and develop coping strategies. The TDAs will provide tailored information, reinforce progress, and review challenges throughout the quit journey. Caregivers may assist with logistics but will not participate in counselling sessions. Participants will receive reimbursement for their travel and time for research purposes (BDT 1000/INR 500/PKR 1500 per visit).

**Fig. 1:**
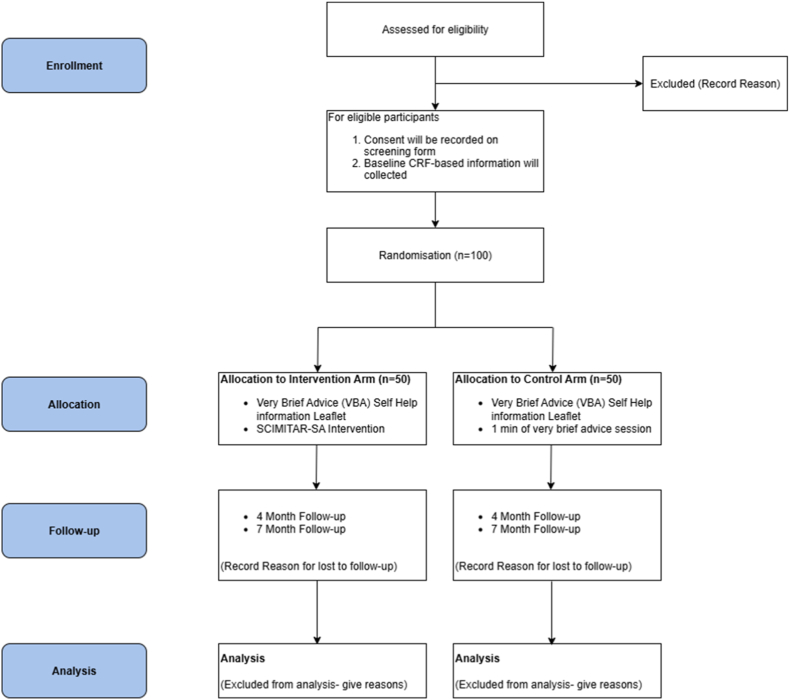
Trial flow diagram.

SCIMITAR-SA is a behavioural programme; cessation aids such as nicotine replacement therapy (NRT) are not provided but may be used if available through usual care. When participants choose to use NRT, TDAs will advise on appropriate type, dosage (quantity and strength), and potential side effects, with details recorded in case report forms at follow-up assessments (see Fig. 1).

### 2.7 Details of the intervention materials

**Fig. 2:**
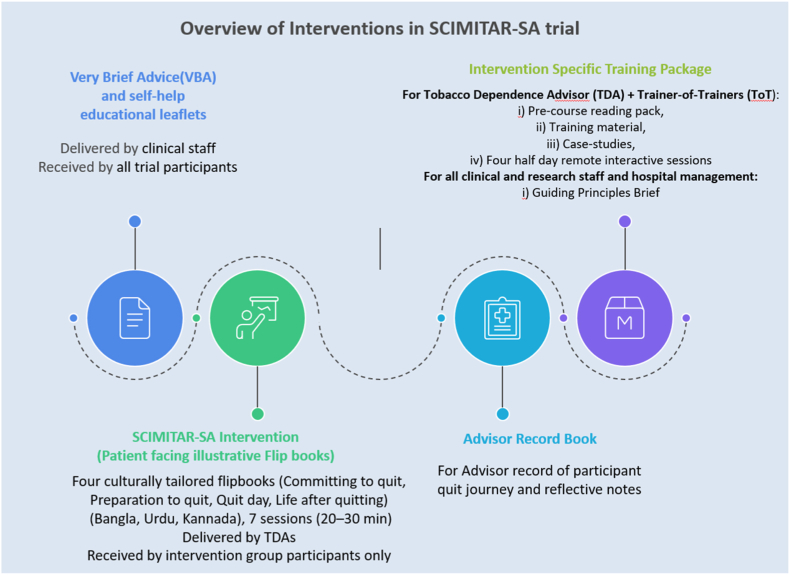
Intervention materials used in SCIMITAR-SA feasibility trial.

The materials that will be used for SCIMITAR-SA trial are described in Fig. 2 below:

### 2.8 Identification and screening of trial participants

At each participating outpatient facility, mental-health professionals (psychiatrists, psychologists, or trainees) will identify tobacco-using individuals with a confirmed SMI diagnosis and provide a VBA + leaflet. Eligible patients will then be referred to Research Assistants (RAs) for screening. Before screening, patients will be asked for permission to be assessed for eligibility. The RA will administer a screening form to determine eligibility. Each trial site will maintain a daily screening log documenting the number of individuals assessed, their eligibility status, and reasons for ineligibility where applicable.

### 2.9 Recruitment of participants for the trial

Eligible individuals will receive a translated Participant Information Sheet (Bangla, Urdu, Kannada, or Hindi) describing the study purpose, procedures, risks, benefits, confidentiality, and voluntary nature of participation. For participants unable to read or write, the information will be read aloud in the presence of a caregiver or impartial witness. Written informed consent will be obtained before any baseline assessment. Consenting participants will sign or provide a thumb impression, countersigned by a witness when required by local ethics committees. Copies of the signed consent form and information sheet will be provided to participants, with originals securely stored at the trial site. Participants may take up to one week to decide; the RA will maintain contact during this period.

Once consented, baseline data will be collected and participants randomised. Those allocated to the intervention arm will be scheduled for counselling sessions by the TDA. Participants preferring to delay consent will be logged for follow-up, and reasons for declining participation will be recorded where possible. Each screened individual will be assigned a unique study ID. Non-identifiable data (age, sex, diagnosis, tobacco-use status, eligibility outcome, and screening duration) will be entered into the (REDCap) [36] database. Identifiable data for consented participants will be stored separately in site-based logs.

Participants may withdraw consent and leave the trial at any time without giving a reason. Written contact details for withdrawal will be provided, and participants may inform any member of the research team if they wish to withdraw. No further data will be collected after withdrawal. Data collected up to that point will be retained for analysis; if data removal is requested, all personally identifiable data will be deleted while anonymised data will be retained and analysed in accordance with GDPR research exemptions.

## 3 Randomisation and trial arm allocation

Participants will be randomly allocated to the intervention or control arm using consecutively numbered, sealed opaque envelopes. The randomisation schedule will be generated by a statistician at the York Trials Unit (YTU) using Stata (version 19) [37] with stratified block randomisation by site and tobacco-use type (smoked only, smokeless only, or dual users), employing variable block sizes of two and four. Each allocation will be printed on a card, sealed in an envelope, and labelled sequentially according to the schedule. Envelopes will be securely stored at each site and accessed only by authorised staff. Following baseline data collection, the RA will contact a trial coordinator at the central research office, who will open the next envelope, record the allocation, and inform the RA. The RA will document the allocation in the baseline e-CRF and notify participants. The coordinator will inform the TDA of intervention-arm participants to schedule intervention sessions.

Web-based or REDCap randomisation will not be used due to unreliable internet connectivity across participating outpatient facilities, which could compromise real-time allocation during clinic workflows. Sealed opaque envelopes will therefore be used as a pragmatic and robust approach suitable for multi-country implementation in these settings. Envelope integrity will be monitored through central oversight, sequential numbering, secure storage, and regular cross-checking of allocation logs against the master randomisation schedule.

### 3.1 Blinding

Blinding of participants, mental health professionals, and research staff is not feasible. However, allocation concealment will be maintained until completion of baseline assessments. To ensure objectivity in outcome assessment, tobacco cessation status will be biochemically verified using saliva cotinine and anabasine testing. Cotinine serves as a biomarker of nicotine exposure, while anabasine differentiates tobacco use from nicotine replacement therapy.

## 4 Baseline and follow-up data collection procedures

After recruitment, data will be collected from participants, with or without the support of caregivers, at baseline (after informed consent and prior to randomisation) and at four and seven months post-randomisation. The trained RAs will administer all instruments in person using REDCap [36] on electronic tablets. Participants in the intervention arm will have a 30-day grace period to set their first quit date, and all follow-up assessments will take place at four and seven months after randomisation.

The information collected at each follow-up, is summarised in Table 1.

**Table 1:** Baseline and follow-up data collection procedures.

	Measurement/data collection	Baseline	4 month follow-up	7 month follow-up
1	Socio-demographic and household information	X		
2	History of tobacco use and attempts to quit	X		
3	Quick SCID (module A and B only)	X		
4	Brief Psychiatric Rating Scale (BPRS)	X		X
5	Self-reported abstinence from tobacco products (past 7-days and since quit date)		X	X
6	Salivary cotinine and anabasine (for biochemical verification)			X
7	Nicotine dependence and urge to use tobacco	X	X	X
8	Quitting intentions, motivations and behaviour	X		
9	Health-related quality of life	X	X	X
10	Mental and physical health	X	X	X
11	Health service utilisation	X	X	X
12	Physical measurements – height and weight, waist circumference Vital signs (Blood pressure, heart rate)	X	X	X
13	Researcher meetings to discuss trial processes	X	X	X
14	Interviews with mental health staff and managers to discuss trial processes and SCIMITAR-SA implementation		X	
15	Focus group discussions with TDAs to discuss SCIMITAR-SA delivery, implementation and administration		x	
16	Interviews with dual users to gather feedback on trial processes and the SCIMITAR-SA intervention		X	
17	Participant session records held by TDAs		X	

**At baseline**, data will be obtained on socio-demographic characteristics (age, gender, education, occupation, marital status, and household assets to determine socioeconomic status using items from the ASTRA trial) [38] and diagnosis of SMI confirmed through the Quick SCID [39]. The psychiatric symptom severity will be assessed at baseline and seven months using the Brief Psychiatric Rating Scale (BPRS) [40]. The history of tobacco use (age at initiation, type, frequency, duration, and prior quit attempts) will be collected using items adapted from the ASTRA [38], Quit4TB and IMPACT-4S [28] trials. Nicotine dependence will be measured using the adapted Heaviness of Smoking Index from ASTRA trial [38], withdrawal symptoms using the Mood and Physical Symptoms Scale (MPSS) [41] and motivation to quit through the Motivation to Quit (MTQ) questionnaire [42]. The EQ-5D-5L [43] will assess health-related quality of life(HRQoL), while the PHQ-9 [44], GAD-7 [45], and SF-12 [46] will measure depressive and anxiety symptoms and physical and mental health status, respectively.

**At four months follow-up**, data collection will focus on tobacco-use outcomes, nicotine dependence, withdrawal symptoms, health-related quality of life, mental and physical health, health-service utilisation, and anthropometric measures. Specifically, tobacco-use status since the quit date and in the previous week will be assessed using items from the ASTRA trial [38], including any use of nicotine products (such as e-cigarettes or nicotine oral pouches). Nicotine dependence will be measured using the adapted Heaviness of Smoking Index from ASTRA trial [38], withdrawal symptoms using the Mood and Physical Symptoms Scale (MPSS) [41]. The EQ-5D-5L [43] will assess health-related quality of life(HRQoL), while the PHQ-9 [44], GAD-7 [45], and SF-12 [46] will measure depressive and anxiety symptoms and physical and mental health status, respectively. Health-service use and out-of-pocket expenditures will be captured using a questionnaire adapted from previous trials (ASTRA [38], POTENTIAL [47], and RESPIRE [48]) and height and weight will be recorded to calculate BMI following WHO protocols [49].

**At seven months follow up,** all measures from the four-month assessment and BPRS will be repeated, and an additional salivary sample will be collected to biochemically verify abstinence. We will operationalise the biochemically verified continuous abstinence from all tobacco products using the global Russell Standard of sustained and verified quitting [50]. For those who report using tobacco products on no more than 5 occasions during the cessation period (duration between the target quit date (for intervention group) or 30 days post-randomisation (for the control group) and the seven month follow up time point) at the seven-month follow-up, biochemical verification will be carried out through salivary cotinine and Anabasine testing through LC-MS (ACM Global Laboratories, UK for Bangladesh and Pakistan & Laboratory of the Department of Clinical Psychopharmacology and Neurotoxicology for India). Cotinine level <15 ng/ml will be used to identify biochemically verified abstinence), whereas those who self-report continuous abstinence but have cotinine levels above the threshold will be identified as tobacco users. Biochemically verified tobacco abstinence at seven months is included as an exploratory outcome to assess the feasibility of collecting cessation outcome data, including biochemical verification, and to inform outcome selection, measurement procedures, and sample size estimation for a future definitive trial.

Follow-up assessments at four and seven months post-randomisation will be considered on time if completed within a one-week window before or after the scheduled date. RAs will begin contacting participants one week prior to, and for up to three weeks after, each due date, making up to three phone calls per week at varying times and days to schedule the assessment. Instances of delayed follow-up will be documented and reviewed after the feasibility phase to refine procedures for the main trial. If the four-month data are missed, efforts will still be made to obtain information at the seven-month follow-up.

## 5 Outcomes

The study will report on the following feasibility outcome measures to address the research questions for the trial:

i) Recruitment, retention, acceptability
  1. Recruitment rates, assessed as the number of participants eligible, consenting and randomised, out of those screened.
  2. Reasons for ineligibility/non-participation/non-consent of participants where provided.
  3. Retention in the study, assessed as the number of participants randomised who are successfully followed up at four and seven months post randomisation with details of withdrawals and loss to follow-up where available.
  4. Qualitative feedback from research staff, health facility staff and trial participants on trial processes
ii) Intervention Delivery
  1. Retention in intervention reported as the total number of sessions attended out of the total number of sessions offered.
  2. Review of participant session records held by TDAs
  3. Qualitative feedback from TDAs, mental health facility staff and trial participants on delivery, implementation and receipt of the SCIMITAR-SA intervention.
iii) Data completion
  1. Completeness of data for baseline assessments, outcome measures for the definitive trial and data on health resource use at four and seven months.
  2. Data completeness of self-reported and biochemically verified continuous abstinence from all tobacco products at seven months.

### 5.1 Sample size

The trial will recruit 100 participants across the six facilities (10 from the two Karachi facilities and 20 from the remaining four) which will allow estimation of recruitment (50%) and retention (80%) to within a 7% and 8% margin of error.

### 5.2 Statistical analysis

No formal statistical comparisons will be undertaken. Continuous variables will be summarised using means and standard deviations, or medians and interquartile ranges where data are skewed, while categorical variables will be reported as frequencies and percentages. A CONSORT [51] flow diagram will illustrate participant progression through screening, recruitment, intervention delivery and follow-up. The numbers screened, eligible, consenting, and recruited will be summarised, including reasons for ineligibility or non-consent where available. For intervention participants, session attendance will be reported, and recruitment and retention rates by trial arm will be presented with 95% confidence intervals(CIs). Data completion rates for each outcome measure will be summarised by trial arm at baseline and follow-up, along with reasons for withdrawal and any reported adverse events. All analyses will be conducted using Stata (version to be confirmed).

### 5.3 Process evaluation

A qualitative process evaluation will be embedded within the feasibility trial to examine (1) the feasibility of trial processes and (2) the delivery of the SCIMITAR-SA intervention and VBA, with a focus on barriers and enablers to implementation within mental-health settings and on providing cessation support to dual users, a novel component of the intervention.

### 5.4 Feasibility of trial processes

Research teams in each country will meet at key stages, i.e., after recruitment, intervention delivery, and follow-up, to reflect on procedures including participant invitation (including reports of cessation advice from referring clinician), consent, intervention delivery, data collection, reimbursement, and retention. Meetings will be audio-recorded, summarised, and later reviewed collectively across countries to identify barriers and enablers and agree process improvements. Feedback from mental-health staff involved in identifying or referring individuals with SMI for the trial will also be explored through interviews. In addition, a subset of dual-user participants will be asked about their experience of recruitment, consent, scheduling, and data-collection procedures during follow-up interviews.

### 5.5 Delivery of the SCIMITAR-SA intervention

Following completion of intervention delivery, fidelity will be examined using participant session records completed by the TDAs (supplementary file 1). The TDAs will then participate in focus group discussions (six in total) to explore experiences of delivery including fidelity to the intervention protocol, supporting dual users, training and supervision, practical challenges in outpatient settings, and administrative issues such as session scheduling and travel reimbursements. Additionally, 6–12 mental-health staff involved in patient referral, VBA delivery, or trial oversight will be interviewed to gather feedback on integration of the intervention within routine services including potential spillover of behavioural techniques into VBA. We will assess the extent and nature of contamination to refine the methods for the definitive trial and to implement any necessary mitigation strategies. A purposive sub-sample of intervention-arm participants who are dual users (approximately 12, two per site) will be interviewed at the four-month follow-up regarding acceptability and feasibility of the intervention; caregivers may join interviews if participants wish.

All interviews and focus groups will be conducted in local languages at study sites, guided by structured topic guides, and audio-recorded with prior informed consent. Interviews will last up to 30 min and focus groups 60–90 min. The recordings will be transcribed, translated into English, and analysed using the Framework Approach [52] to address applied implementation questions. Microsoft Excel will be used to support data management and analysis.

### 5.6 Economic analysis

The economic analysis will involve a preliminary assessment of healthcare resource use and intervention costs from the national health system perspective. Costs of delivering SCIMITAR-SA will be estimated by identifying resources required for set-up, training, supervision, and delivery, including staff time, materials, nicotine replacement therapy (where applicable), communication costs, information sheets, client record materials, and overheads. Comparator costs will include only those related to delivering VBA, typically lasting up to 1 min [53]. Healthcare utilisation data will be collected at baseline and follow-up using self-reported service-use questionnaires, which will be refined based on feasibility findings. During the feasibility stage, patient-level cost profiles and full cost-effectiveness analyses will not be conducted due to limited sample size; instead, intervention costs, healthcare utilisation, and EQ-5D-5L [43] outcomes will be summarised descriptively by trial arm. The analysis will assess the practicality of collecting comprehensive economic and quality of life data and the feasibility of conducting a full economic evaluation alongside a definitive trial.

### 5.7 Data management

Two secure REDCap databases will be maintained for the feasibility trial, by ARK Foundation (Bangladesh and Pakistan) and NIMHANS (India). Data managers at each site will perform standardised quality checks, and only authorised personnel (principal investigators, country investigators, coordinators, and research fellows) will have access to identifiable data, held securely at the respective country site and used solely to facilitate participant follow-up. Prior to analysis all data will be anonymised, identified only using unique study IDs.Electronic data will be captured via an encrypted REDCap mobile app [54], stored on secure servers at ARK and NIMHANS, and shared with the University of York for analysis via secure Drop-off services. Qualitative recordings and transcripts will be anonymised, stored in password-protected folders, and erased from recorders after transcription. Separate encrypted files linking personal identifiers with study IDs will be securely maintained. The ARK team will develop a recruitment dashboard and ensure data integrity, preventing duplication or loss. If a participant withdraws consent, only identifiable data will be deleted, while anonymised study data will be retained. All sites will comply with General Data Protection Regulation(GDPR) [55], the UK Data Protection Act (2018) [36], and any local regulations of partner countries. Study materials, including consent forms, screening logs, and anonymised transcripts, will be accessible only to authorised researchers or monitors. At trial completion, anonymised datasets will be archived by the University of York for at least 10 years in line with UK Good Clinical Practice (GCP) guidelines [56] before secure destruction. Hard-copy study documents at all sites, coordinating centres, and the University of York will be stored in locked, secure locations during and after the trial.

### 5.8 Trial data quality and ethical considerations

Data quality will be maintained through standardised procedures and oversight by data managers at ARK Foundation and NIMHANS, with additional verification by statisticians at ARK and the University of York. Study data will be securely captured on REDCap servers, de-identified, and password-protected, with restricted editing rights. Automated audit trails will record all data changes, and final datasets will be validated, cleaned, and locked before analysis. Summary reports will be shared periodically with the Independent Advisory Board (IAB).

The SCIMITAR-SA feasibility trial involves individuals with SMI, caregivers, and healthcare staff, and will include sensitive health data sharing across countries. Key ethical considerations include informed consent, data confidentiality, participant vulnerability, and equitable engagement. Participation will require demonstrated capacity to consent, with the right to withdraw at any time. The intervention is low-risk and participant burden will be minimised. Community contributors will be appropriately recognised per NIHR guidance. Ethical oversight will be provided by the Programme Management Group and IAB, with data handled under ARK and University of York governance policies. Staff and participant safety will be ensured through site-specific risk assessments and safeguarding procedures, and participant materials will include independent ethics contact details.

### 5.9 Adverse events

Adverse events (AEs) and serious adverse events (SAEs) will be identified, recorded, and reported in accordance with the ICH-GCP [57,58] and national regulations. Although SCIMITAR-SA is a behavioural intervention with minimal anticipated risks, potential AEs include psychological distress during data collection, NRT side effects, and exacerbation of existing mental illness. Participants will be informed about possible side effects and encouraged to report any AEs via study contacts. RAs will assess AEs during counselling sessions and at four- and seven-month follow-ups using standardised checklists and case report forms. Suicidal ideation or self-harm risk will be managed using a suicide-risk pathway adapted from the DiaDeM research programme [59], with immediate referral to designated mental-health professionals. In the event of an exacerbation of mental illness or the occurrence of adverse effects related to NRT, participants will be referred for consultation with their treating psychiatrist. Each AE will be assessed for seriousness, severity, causality, and expectedness. Serious or unexpected events related to the intervention will be reported within 24 h to country leads and within seven days to the University of York, which will coordinate onward reporting to ethics and regulatory authorities. All events will be followed to resolution. Safety data will be summarised descriptively, with all AEs and SAEs promptly reported. Annual safety reports and urgent safety measures will be managed by the sponsor (University of York) in collaboration with country leads.

### 5.10 Trial audit and management

Someone external to the SCIMITAR-SA research team in each country will conduct monthly site visits using standardised checklists to verify adherence to the protocol, SOPs, and documentation standards. A lead investigator will complete at least one scheduled monitoring visit per country, with unscheduled visits by trial data coordinators if data quality or compliance concerns arise or if logistical issues require on-site resolution.

The Programme Management Group (PMG), comprising all study investigators, will meet monthly to oversee delivery, analysis, and dissemination. An Independent Advisory Board (IAB), consisting of experts in tobacco control, mental health, or global health research chaired by an external independent expert, will meet twice in the first year and annually thereafter. The IAB may recommend halting the trial if new evidence indicates harm or benefit, though early benefits alone will not stop recruitment.

### 5.11 Progression criteria to a full trial

Progression from feasibility to the full trial will be guided by the Independent Advisory Board (IAB) using a “traffic light” framework.

**Green (proceed):** ≥80% recruitment within the target period, ≥80% retention at seven months (<10% difference between arms), and ≥80% completeness of primary outcome data (self-reported smoking status with biochemical verification).

**Amber (proceed with amendments):** Recruitment, retention, or data completeness of 50–79%, or minor procedural issues requiring adjustment.

**Red (review required):** <50% recruitment or retention, >16% difference between arms, or <50% outcome data availability.

Acceptability and feasibility of trial procedures and intervention delivery will also inform progression, assessed qualitatively through feedback from participants, TDAs, mental-health staff, and research teams.

### 5.12 Dissemination of trial findings

Findings will be published in peer-reviewed journals following SCIMITAR-SA's publication policy and ICMJE guidelines [60], and presented at international meetings on tobacco control, psychiatry, and global health. Summaries will be shared with participants, caregivers, and communities through reports and local dissemination by Community Advisory Panels (CAPs) in each country. Wider visibility will be achieved via media, the SCIMITAR-SA website(https://www.impactsouthasia.com/scimitar-sa/) and official LinkedIn (Scimitar South Asia), Instagram(@Scimitar_SA),and Threads (@Scimitar_SA) accounts, especially around World Mental Health and World No Tobacco Days.

## 6 Discussion

The SCIMITAR-SA feasibility trial will address a critical evidence gap by evaluating the feasibility of a culturally relevant behavioural intervention to support tobacco cessation among individuals with SMI in South Asia. This population faces high rates of tobacco dependence and significant barriers to cessation, yet tailored, evidence-based support remains limited. The study's participant-centred approach integrates cessation support for smokers, smokeless tobacco (SLT) users, and dual users, combining behavioural intervention with optional NRT (where available as part of routine care) delivered within existing mental health services.

A major strength of the study lies in its multi-country implementation across diverse health-system contexts in Bangladesh, India, and Pakistan, using consistent, validated tools and a robust data-collection framework. The inclusion of qualitative process-evaluation components adds depth to understanding contextual barriers and facilitators of implementation. Moreover, the focus on integrating cessation support into routine psychiatric care highlights the feasibility of sustainable delivery models.

However, as a feasibility study with a modest sample size, it is not powered to detect significant differences in effectiveness outcomes. Other limitations include potential variability in NRT availability across sites, challenges in retaining a highly clinically vulnerable population, and possible under-reporting of tobacco use due to social desirability bias. Despite these constraints, SCIMITAR-SA provides an essential foundation for a definitive trial and strengthens the evidence base for integrating tobacco cessation into mental health care in low- and middle-income settings.

### 6.1 Protocol amendments

The current version of the trial protocol (Version 3.0, dated August 20, 2025) incorporates revisions recommended by the trial sponsor and the Trial Management Team (PMG and IAB) to date. All proposed amendments are reviewed with the Chief Investigators before submission to the University of York Research Governance Committee (HSRGC) for formal consideration. The Committee determines whether each amendment is classified as major or minor, following institutional guidance. Minor amendments are implemented upon notification, whereas major amendments require prior approval from the respective national ethics committees in each participating country before implementation.

## CRediT authorship contribution statement

**Garima Bhatt:** Writing – review & editing, Writing – original draft, Project administration, Conceptualization. **Kousar Ishaq:** Writing – review & editing, Project administration, Investigation, Conceptualization. **Aatik Arsh:** Writing – review & editing, Resources, Project administration, Methodology, Conceptualization. **Omara Dogar:** Writing – review & editing, Visualization, Resources, Funding acquisition, Conceptualization. **Justin Fenty:** Writing – review & editing, Validation, Formal analysis, Conceptualization. **Cath Jackson:** Writing – review & editing, Resources, Conceptualization. **Muqaddas Asif:** Writing – review & editing, Resources, Investigation, Conceptualization. **Jothisha C:** Writing – review & editing, Resources, Investigation, Data curation, Conceptualization. **Shannon Halmkan:** Writing – review & editing, Validation, Formal analysis, Data curation, Conceptualization. **Fahmida Islam:** Writing – review & editing, Resources, Investigation, Conceptualization. **Krishna Prasad Muliyala:** Writing – review & editing, Methodology, Funding acquisition, Conceptualization. **Venkata Lakshmi Narasimha:** Writing – review & editing, Resources, Funding acquisition, Conceptualization. **Steve Parrott:** Writing – review & editing, Visualization, Funding acquisition, Conceptualization. **Rebecca R:** Writing – review & editing, Resources, Investigation, Conceptualization. **Fahmidur Rahman:** Software, Data curation, Conceptualization. **Faraz Siddiqui:** Writing – review & editing, Supervision, Methodology, Funding acquisition, Conceptualization. **Heather Thomson:** Writing – review & editing, Resources, Conceptualization. **Han-I Wang:** Writing – review & editing, Conceptualization. **Rumana Huque:** Writing – review & editing, Methodology, Funding acquisition, Conceptualization. **Imran B. Chaudhry:** Methodology, Funding acquisition, Conceptualization. **Asad Tamizuddin Nizami:** Methodology, Funding acquisition, Conceptualization. **Catherine Hewitt:** Visualization, Methodology, Funding acquisition, Conceptualization. **Najma Siddiqi:** Writing – review & editing, Supervision, Project administration, Methodology, Conceptualization. **Kamran Siddiqi:** Writing – review & editing, Supervision, Project administration, Methodology, Funding acquisition, Conceptualization. **Simon Gilbody:** Visualization, Supervision, Project administration, Methodology, Conceptualization. **Pratima Murthy:** Visualization, Supervision, Methodology, Funding acquisition, Conceptualization.

## Availability of data and materials

Not applicable.

## Ethics approval and consent to participate

The feasibility trial protocol has been approved by the Health Sciences Research Governance Committee (HSRGC) at the University of York (Ref: HSRGC/2024/626/E: SCIMITAR-SA), National Research and Bioethics Committees in Bangladesh (National Research Ethics Committee (NREC) functioning under Bangladesh Medical Research Council (BMRC)), (Ref: BMRC/NREC/2025-2027/128), Health Ministry's Screening Committee (HMSC) operated by the Department of Health Research/Indian Council of Medical Research (ICMR)) and the Screening Committee for Research Proposal (SCRP), Department of Health and Family Welfare, Ministry of Health and Family Welfare, Government of India (Ref: F.No P- 29010/19/2025-DMCell), Ethics Committee (Behavioural Science Division) of National Institute of Mental Health and Neurosciences, Bengaluru, India, (Ref: NIMHANS/47th IEC (BEH.SC.DIV.)/2024), National Bioethics Committee for Research (NBC-R) at Health Research Institute, National Institutes of Health (NIH)) in Pakistan (Ref: 4-87/NBCR-1145/24-25/1422), and Institutional Research & Ethics Forum (IREF), Rawalpindi Medical University (RMU), Rawalpindi, Pakistan (Ref: 810/IREF/RMU/2024). Any amendment to the protocol will be submitted for approval to the same ethics governing bodies in respective partner countries. The informed consent to participate in the feasibility trial will be obtained directly from the patient(s).

## Consent for publication

Not applicable.

## Funding

The SCIMITAR-10.13039/100012106SA programme is funded by the 10.13039/501100000272National Institute for Health and Care Research [Grant reference: Research & Innovation for Global Health Transformation (RIGHT) NIHR205601] and is sponsored by the 10.13039/100009001University of York, UK. The views expressed in the manuscript are of the authors and not necessarily those of the NIHR or the UK Department of Health and Social Care.

## Declaration of competing interest

The authors declare that they have no known competing financial interests or personal relationships that could have appeared to influence the work reported in this paper.

## Data Availability

No data was used for the research described in the article.

## Appendix A Supplementary data

Supplementary file 1 Patient Record Sheet. Supplementary file 1

Supplementary file 2 SPIRIT Checklist: Recommended items to address in a randomised trial protocol. Supplementary file 2

Supplementary file 3 TIDieR checklist. Supplementary file 3
