# Supplementary material for: Tobacco cessation intervention for individuals with severe mental illness in Bangladesh, India, and Pakistan: protocol for a multi-country feasibility randomised controlled trial (SCIMITAR-SA)
Source: Contemp Clin Trials Commun. 2026 Feb 21;50:101620. doi: 10.1016/j.conctc.2026.101620 (PMC12969440; doi:10.1016/j.conctc.2026.101620)
Supplement: Supplementary file 1 — Patient Record Sheet. [file mmc1.docx]

Patient record sheet

| Name | Trial Reg. no | Contact details (phone only) | Name of facility |
| --- | --- | --- | --- |
|  |  |  |  |

ADMINISTRATION RECORD

**Phone calls to schedule the session**

| **Appointment number** | **Title of appointment**  Record as:   - **C2Q** =Committing to quit - **P2Q**= Preparing to quit - **QD**=Quit day - **PQ1**= Post quit session 1 - **PQ2**= Post quit session 2 - **PQ3**= Post quit session 3 - **PQF**= Final post quit session | **Number of attempts to make contact** | **Date of actual contact:**  Record as:  **DD/MM/YYYY** | **Outcome of contact:**  Record as:   - **DNW**=Did not want to book appointment - **B DD/MM/YYYY Method of delivery** =Booked new appointment, date of that appointment and planned method of delivery - **RB DD/MM/YYYY** **Method of delivery** =Re-booked missed appointment and date of that appointment and planned method of delivery - **Other** (please state)   **Codes for method of delivery**   - **F2F**=Face to Face - **RT**=Remote by telephone - **RV**=Remote by video call - **Other** (please state) | **Any challenges with scheduling this appointment**  Record as: yes/no  **If yes, what were the challenges?** |
| --- | --- | --- | --- | --- | --- |
|  |  |  |  |  |  |
|  |  |  |  |  |  |
|  |  |  |  |  |  |
|  |  |  |  |  |  |

**Delivery of session**

| **Appointment number** | **Title of session**  Record as:   - **C2Q** =Committing to quit - **P2Q**= Preparing to quit - **QD**=Quit day - **PQ1**= Post quit session 1 - **PQ2**= Post quit session 2 - **PQ3**= Post quit session 3 - **PQF**= Final post quit session | **Date of**  **appointment**  Record as:  **DD/MM/YYYY** | **Duration of appointment**  Record the number of minutes | **Method of delivery**  Record as:   - **F2F**=Face to Face - **RT**=Remote by telephone - **RV**=Remote by video call - **Other** (please state) | **Outcome of appointment**  Record as:   - **A**=Attended - **DNA-apology**=Did not attend but told TDA - **DNA**= Did not attend and did not tell the TDA - **DIS =** disengaged with the cessation programme - **Other** (please state) | **Did a caregiver attend the appointment?**  Record as: yes/no  **If yes, who attended?** | **Any challenges with delivering this appointment?**  Record as: yes/no  **If yes, what were the challenges?**  **(include details of splitting sessions and reason for this)** |
| --- | --- | --- | --- | --- | --- | --- | --- |
|  |  |  |  |  |  |  |  |
|  |  |  |  |  |  |  |  |
|  |  |  |  |  |  |  |  |
|  |  |  |  |  |  |  |  |
|  |  |  |  |  |  |  |  |
|  |  |  |  |  |  |  |  |
|  |  |  |  |  |  |  |  |
|  |  |  |  |  |  |  |  |
|  |  |  |  |  |  |  |  |
|  |  |  |  |  |  |  |  |
|  |  |  |  |  |  |  |  |

FLIPBOOK DELIVERY RECORD

**NOTE: For patients who are stopping using SMOKED tobacco products (e.g. cigarettes / bidis)**

**Is the patient taking any medications that may require dose adjustment because of stopping smoking?** □ Yes □ No

**Ensure the prescribing clinician is aware that the patient is attempting to stop smoking and provide the quit date when known.**

**Ensure any change in smoking status during the programme is communicated to the prescribing clinician.**

**General notes as a reminder:**

e.g. preferred name / title / occupation / additional information about smoking history etc.

**Flip Book 1 – Committing to Quit**

| FB ref |  |  | | | | |
| --- | --- | --- | --- | --- | --- | --- |
| 1.2 | Key reasons for using tobacco | □ Improved mood  □ Improved concentration  □ Stress / anxiety relief  Other: | | | | |
| 1.4 | Key benefits of stopping using tobacco | □ Improved health  □ Save money  □ Social benefits  □ Impact on family  Other: | | | | |
|  | Top 3 reasons to quit – please note | 1)  2)  3) | | | | |
| 1.5 | Current tobacco use | Main Products used | How often per day | | How much per day | |
|  |  | 1. |  | |  | |
|  |  | 2. |  | |  | |
|  |  | 3. |  | |  | |
| 1.6 | Any previous quit attempts | □ Yes □ No  Comments: | | | | |
| 1.7 | Importance score (please circle) | Initial score | 1 | 2 | | 3 |
|  |  | Amended score | 1 | 2 | | 3 |
| 1.8 | Confidence score | Initial score | 1 | 2 | | 3 |
| 1.9 | Any concerns about quitting | □ Yes □ No  Comments: | | | | |
| 1.10 | Support from friend / family member | □ Yes □ No  Comments: | | | | |
| 1.13 | Confidence score | Amended score | 1 | 2 | | 3 |
| Any further information: | | | | | | |

**Flip Book 2 – Preparing to Quit**

| FB ref |  |  | | | |
| --- | --- | --- | --- | --- | --- |
| 2.1 | Current confidence score (please circle) | 1 | 2 | | 3 |
| 2.2 | Heaviness of tobacco use index score *(smoked tobacco products)* | On the days that you smoke, how soon after waking up do you have your first (*cigarette)*? | | 1. Within 5 minutes (3 points) 2. 6-30 minutes (2 points) 3. 31-60 minute (1 point) 4. After 60 minutes (0 points) | |
|  |  | How many (*cigarettes)* do you typically smoke per day? | | 1. 10 or fewer (0 points) 2. 11-20 (1 point) 3. 21-30 (2 points) 4. 31 or more (3 points) | |
|  | Heaviness of tobacco use index score *(smokeless tobacco products)* | On the days that you use tobacco, how soon after waking up do you have your first *(paan/gutka/naswar)*? | | 1. Within 5 minutes (3 points) 2. 6-30 minutes (2 points) 3. 31-60 minute (1 point) 4. After 60 minutes (0 points) | |
|  |  | How many times a day do you take tobacco? | | 1. 10 or fewer (0 points) 2. 11-20 (1 point) 3. 21-30 (2 points) 4. 31 or more (3 points) | |
| 2.4 | Is the patient planning to use NRT Gum  (guide 2mg per cigarette / chew per day)  Or  other nicotine delivery device (e cigarette / pouches etc) | □ Yes □ No  If Yes, Is the patient  □ being provided with NRT gum  □ purchasing their own NRT gum  □ purchasing their own e cigarette / vape  □ purchasing their own nicotine pouches (not containing tobacco)  □ other (please state below) | | | |
| 2.6 | Quit day set | □ Yes □ No  Planned quit date:  Planned date and time of last cigarette/chew: | | | |
| 2.7 | Personal statement | Please note the personal statement: | | | |
| Any further information: | | | | | |

**Flip Book 3 – Quit Day**

| FB ref |  |  | |
| --- | --- | --- | --- |
| 3.3 | Have any withdrawal symptoms been experienced so far | □ Yes □ No  □ Urges to smoke / use tobacco  □ Sadness / depression  □ Restlessness / Poor concentration  □ Irritability / aggression  □ Other (please describe) | |
|  | How have these been managed? |  | |
| 3.4 | Key triggers for tobacco use (e.g. doing what, with who, how were you feeling) and changing routines | Trigger | Coping Plan |
|  |  |  |  |
|  |  |  |  |
|  |  |  |  |
|  |  |  |  |
| 3.5 | Does the patient live / work / spend a lot of time with others who use tobacco? | □ Yes □ No  Comments: | |
|  | Has the patient actively sought support from friends /family? Who? | □ Yes □ No  Comments: | |
|  | Have friends / family / others been supportive? | □ Yes □ No  Comments: | |
| 3.6 | Does the patient anticipate any difficult situations in the next week | Situation | Coping Plan |
|  |  |  |  |
|  |  |  |  |
|  |  |  |  |
|  |  |  |  |
| 3.7 | Has the patient set any small targets (e.g. not using tobacco for a day) and thought of a reward? | Target | Reward |
|  |  |  |  |
|  |  |  |  |
|  |  |  |  |

**Flip Book 4 – Life After Quitting week1**

| FB ref |  |  | |
| --- | --- | --- | --- |
| 4.1 | Is the patient tobacco free? | □ Yes, totally tobacco free  □ Nearly but some lapses □a few puffs □ between 1-5 □> 5  □ No, cut down but not stopped *(inform prescribing clinician)*  □ No, still using some tobacco daily *(inform prescribing clinician)*  □ No, not tried to quit *(inform prescribing clinician)* | |
|  | If the goals in 3.7 were achieved, did the patient reward themselves? | □ Yes  □ No  Comment: | |
| 4.2  Only if a product is being used. | Is the patient still using a nicotine replacement product? | □ Yes, supplied to them  □ Yes, purchased by the patient  □ No longer using due to cost  □ No longer using due to side effects / not liking the product | |
|  | Which nicotine replacement product is the patient using? | Please describe: | |
|  | How much nicotine replacement is the patient using each day? | Please describe: | |
| 4.3 | Have any cravings or withdrawal symptoms been experienced this week? | □ Cravings / Urges to smoke / use tobacco  □ Sadness / depression  □ Restlessness / Poor concentration  □ Irritability / aggression  □ Difficulty sleeping  □ Increased appetite / weight gain  □ Other (please describe) | |
|  | Are cravings or withdrawal symptoms becoming less intense? | Cravings / urges to use tobacco  □ Yes  □ No  □ Not sure | Withdrawal symptoms  □ Yes  □ No  □ Not sure |
|  | Is the patient feeling any benefits to stopping? | Please describe: | |
| 4.4 | Has the patient coped with high-risk situations in the **last** week? | Situation | Coping Plan |
|  |  |  |  |
|  |  |  |  |
|  |  |  |  |
|  | Does the patient anticipate any high-risk situations in the **next** week? |  |  |
|  |  |  |  |
|  |  |  |  |

**Flip Book 4 – Life After Quitting week 2**

| FB ref |  |  | |
| --- | --- | --- | --- |
| 4.1 | Is the patient tobacco free? | □ Yes, totally tobacco free  □ Nearly but some lapses □a few puffs □ between 1-5 □> 5  □ No, cut down but not stopped *(inform prescribing clinician)*  □ No, still using some tobacco daily *(inform prescribing clinician)*  □ No, not tried to quit *(inform prescribing clinician)* | |
| 4.2  Only if a product is being used. | Is the patient still using a nicotine replacement product? | □ Yes, supplied to them  □ Yes, purchased by the patient  □ No longer using due to cost  □ No longer using due to side effects / not liking the product | |
|  | Which nicotine replacement product is the patient using? | Please describe: | |
|  | How much nicotine replacement is the patient using each day? | Please describe: | |
| 4.3 | Have any cravings or withdrawal symptoms been experienced this week? | □ Cravings / Urges to smoke / use tobacco  □ Sadness / depression  □ Restlessness / Poor concentration  □ Irritability / aggression  □ Difficulty sleeping  □ Increased appetite / weight gain  □ Other (please describe) | |
|  | Are cravings or withdrawal symptoms becoming less intense? | Cravings / urges to use tobacco  □ Yes  □ No  □ Not sure | Withdrawal symptoms  □ Yes  □ No  □ Not sure |
|  | Is the patient feeling any benefits to stopping? | Please describe: | |
| 4.4 | Has the patient coped with high-risk situations in the **last** week? | Situation | Coping Plan |
|  |  |  |  |
|  |  |  |  |
|  |  |  |  |
|  | Does the patient anticipate any high-risk situations in the **next** week? |  |  |
|  |  |  |  |
|  |  |  |  |

**Flip Book 4 – Life After Quitting week 3**

| FB ref |  |  | |
| --- | --- | --- | --- |
| 4.1 | Is the patient tobacco free? | □ Yes, totally tobacco free  □ Nearly but some lapses □a few puffs □ between 1-5 □> 5  □ No, cut down but not stopped *(inform prescribing clinician)*  □ No, still using some tobacco daily *(inform prescribing clinician)*  □ No, not tried to quit *(inform prescribing clinician)* | |
| 4.2  Only if a product is being used. | Is the patient still using a nicotine replacement product? | □ Yes, supplied to them  □ Yes, purchased by the patient  □ No longer using due to cost  □ No longer using due to side effects / not liking the product | |
|  | Which nicotine replacement product is the patient using? | Please describe: | |
|  | How much nicotine replacement is the patient using each day? | Please describe: | |
| 4.3 | Have any cravings or withdrawal symptoms been experienced this week? | □ Cravings / Urges to smoke / use tobacco  □ Sadness / depression  □ Restlessness / Poor concentration  □ Irritability / aggression  □ Difficulty sleeping  □ Increased appetite / weight gain  □ Other (please describe) | |
|  | Are cravings or withdrawal symptoms becoming less intense? | Cravings / urges to use tobacco  □ Yes  □ No  □ Not sure | Withdrawal symptoms  □ Yes  □ No  □ Not sure |
|  | Is the patient feeling any benefits to stopping? | Please describe: | |
| 4.4 | Has the patient coped with high-risk situations in the **last** week? | Situation | Coping Plan |
|  |  |  |  |
|  |  |  |  |
|  |  |  |  |
|  | Does the patient anticipate any high-risk situations in the **next** week? |  |  |
|  |  |  |  |
|  |  |  |  |

**Flip Book 4 – Life After Quitting week 4**

| FB ref |  |  | |
| --- | --- | --- | --- |
| 4.6 | Is the patient tobacco free? | □ Yes, totally tobacco free since quit day  □ Yes, tobacco free for the last 2 weeks, but some lapses in first 2 weeks after quit day  □ No, still using tobacco daily *(inform prescribing clinician)* | |
| 4.7  Only if a product is being used. | Is the patient still using a nicotine replacement product? | □ Yes, supplied to them  □ Yes, purchased by the patient  □ No longer using due to cost  □ No longer using due to side effects / not liking the product | |
|  | Which nicotine replacement product is the patient using? | Please describe: | |
|  | How much nicotine replacement is the patient using each day? | Please describe: | |
| 4.8 | Have any cravings or withdrawal symptoms been experienced this week? | □ Cravings / Urges to smoke / use tobacco  □ Sadness / depression  □ Restlessness / Poor concentration  □ Irritability / aggression  □ Difficulty sleeping  □ Increased appetite / weight gain  □ Other (please describe) | |
|  | Are cravings or withdrawal symptoms becoming less intense? | Cravings / urges to use tobacco  □ Yes  □ No  □ Not sure | Withdrawal symptoms  □ Yes  □ No  □ Not sure |
|  | Is the patient feeling any benefits to stopping? | Please describe: | |
| 4.9 | Has the patient coped with high-risk situations in the **last** week? | Situation | Coping Plan |
|  |  |  |  |
|  |  |  |  |
|  |  |  |  |
|  | Does the patient anticipate any high-risk situations in the **future**? |  |  |
|  |  |  |  |
|  |  |  |  |
